# Diagnostic Value of Detecting Feline Coronavirus RNA and Spike Gene Mutations in Cerebrospinal Fluid to Confirm Feline Infectious Peritonitis

**DOI:** 10.3390/v13020186

**Published:** 2021-01-27

**Authors:** Sandra Felten, Kaspar Matiasek, Christian M. Leutenegger, Laura Sangl, Stephanie Herre, Stefanie Dörfelt, Andrea Fischer, Katrin Hartmann

**Affiliations:** 1 Clinic of Small Animal Medicine, Center for Clinical Veterinary Medicine, LMU Munich, Veterinärstr. 13, 80539 Munich, Germany; laura.sangl@gmx.de (L.S.); steffi@herre.at (S.H.); s.doerfelt@medizinische-kleintierklinik.de (S.D.); andreafischer@lmu.de (A.F.); hartmann@lmu.de (K.H.); 2 Section of Clinical & Comparative Neuropathology, Center for Clinical Veterinary Medicine, LMU Munich, Veterinärstr. 13, 80539 Munich, Germany; matiasek@patho.vetmed.uni-muenchen.de; 3 IDEXX Laboratories, Inc., 2825 KOVR Dr., West Sacramento, CA 95605, USA; Christian.Leutenegger@antechmail.com

**Keywords:** FCoV, diagnosis, PCR, RT-PCR, FIPV, neurological signs, central nervous system (CNS), discriminative

## Abstract

Background: Cats with neurologic feline infectious peritonitis (FIP) are difficult to diagnose. Aim of this study was to evaluate the diagnostic value of detecting feline coronavirus (FCoV) RNA and spike (S) gene mutations in cerebrospinal fluid (CSF). Methods: The study included 30 cats with confirmed FIP (six with neurological signs) and 29 control cats (eleven with neurological signs) with other diseases resulting in similar clinical signs. CSF was tested for FCoV RNA by 7b-RT-qPCR in all cats. In RT-qPCR-positive cases, S-RT-qPCR was additionally performed to identify spike gene mutations. Results: Nine cats with FIP (9/30, 30%), but none of the control cats were positive for FCoV RNA in CSF. Sensitivity of 7b-RT-qPCR in CSF was higher for cats with neurological FIP (83.3%; 95% confidence interval (95% CI) 41.8–98.9) than for cats with non-neurological FIP (16.7%; 95% CI 6.1–36.5). Spike gene mutations were rarely detected. Conclusions: FCoV RNA was frequently present in CSF of cats with neurological FIP, but only rarely in cats with non-neurological FIP. Screening for spike gene mutations did not enhance specificity in this patient group. Larger populations of cats with neurological FIP should be explored in future studies.

## 1. Introduction

Feline coronaviruses (FCoV) are enveloped RNA viruses belonging to the genus *Alphacoronavirus* within the family *Coronaviridae*. These viruses exist as two serotypes, with serotype I being more prevalent in most countries and serotype II most likely having developed as recombination between serotype I FCoV and canine coronavirus [1]. FCoV are ubiquitous among the cat population and efficiently spread via the fecal-oral route. Usually, infections remain asymptomatic or only cause mild diarrhea [2]. In a small percentage of cats, however, feline infectious peritonitis (FIP) develops secondary to mutation of non-pathogenic FCoV of serotype I or II to a highly pathogenic biotype within an infected cat [3,4]. This biotype, in contrast to non-pathogenic FCoV with a tropism for enterocytes, is capable of effectively replicating in macrophages, thereby leading to macrophage activation and a polysystemic inflammatory state and immune dysregulation [1,5]. Affected cats develop a variety of clinical signs, such as anorexia, lethargy, weight loss, pyrexia, ocular and neurological signs. These abnormalities, however, are rather non-specific. This is also the case for possible clinicopathological changes, including anemia, microcytosis, lymphopenia, neutrophilia, thrombocytopenia, hyperglobulinemia, hypoalbuminemia and hyperbilirubinemia [6].

FIP is considered to be the most common infectious disease of the central nervous system (CNS) in cats [7,8], and up to 30% of cats with FIP are affected by neurological involvement [9,10,11,12]. Typical lesions involve both the brain and spinal cord and usually are surface-related and multifocal. They include meningitis, choroid plexitis, ependymitis, and periventriculitis, which can lead to increase of cerebrospinal fluid (CSF) viscosity and obstructive hydrocephalus or hydromyelia [10,13,14,15,16]. Depending on their localization, these lesions can manifest clinically as seizures, ataxia, hyperesthesia, cranial nerve deficits, central vestibular signs, or behavioral changes [9,10,15,16,17,18,19]. CSF changes such as neutrophilic or pyogranulomatous pleocytosis and elevated protein concentration are common [10,17,20], but analysis can as well be unremarkable [16]. Presence of typical CSF changes increase suspicion of FIP, but findings are not diagnostic for FIP and can also be seen with other inflammatory CNS diseases [20,21] and in hypertensive encephalopathy with disruptive vascular disease [22].

FIP was long considered a uniformly fatal disease, leading to death or euthanasia within a few days after initial presentation. Thus, definitive ante mortem diagnosis was crucial in order to avoid euthanasia in unaffected cats. Recently, novel antiviral drugs have been discovered that lead to clinical cure of cats with FIP [23,24,25,26,27], but these drugs are not yet commercially available and likely expensive, again highlighting the need for definitive diagnosis in order to correctly identify cats that are suitable candidates for treatment. Ante mortem diagnosis, however, still is challenging since non-mutated and mutated FCoV cannot be distinguished reliably by available diagnostic methods [6]. If effusion is present in cats suspected to have FIP, there are a number of possible diagnostic tests with generally better predictive values than tests using blood [6]. However, since neurological signs of FIP occur in up to 43% of cats without effusion compared to only up to 10% cats with effusion [28,29], different diagnostic approach is needed in these cats. The detection of antibodies does not allow a definitive diagnosis of FIP, as they are also present in the serum and even in the CSF of cats with other neurologic diseases [30]. Likely, such false positive CSF antibody test results occur as a result of an impaired blood-brain barrier (BBB) in inflammatory CNS diseases other than FIP and subsequent leakage of antibodies into the CSF [31]. Immunostaining of the FCoV nucleocapsid within macrophages in the CSF of cats suspected of having neurologic or non-neurologic FIP, while providing acceptable sensitivity, can also produce false positive results [32] and the same is true for immunocytochemistry using aqueous humor and fine-needle-aspirates (FNA) of mesenteric lymph nodes [33,34]. FNA and subsequent immunocytochemistry to detect FCoV antigen within the liver and kidneys only has very low sensitivity [35]. Additionally, the detection of feline coronavirus (FCoV) RNA by realtime reverse transcriptase polymerase chain reaction (RT-qPCR) in blood is not useful for a definitive diagnosis either. FCoV viremia can occur in both cats with FIP and healthy FCoV-infected cats that never develop FIP [36], and additionally, sensitivity of RT-qPCR in blood samples is low due to a very low virus load in virtually any blood fraction [37]. If mesenteric lymphadenomegaly is present in a cat suspected of having FIP, RT-qPCR of mesenteric lymph node FNA is possible and was shown to have good sensitivity and specificity [38]. However, small size of the lymph nodes, especially in the typically young cats with FIP might be a limiting factor to this diagnostic approach. Although the specificity of RT-qPCR in CSF was good in previously performed studies [16,39,40], false positive results are still possible if either free or cell-bound FCoV—as suspected for antibodies—pass an impaired BBB in cats with neurologic diseases other than FIP and consequently appear in the CSF. Little is known about the sensitivity of RT-qPCR in CSF of cats with neurologic FIP.

Mutations in the FCoV spike (S) gene and corresponding amino acid substitutions in the S protein are thought to be a crucial element in FIP pathogenesis. Since the S protein contains both subunits required for host cell entry, the acquisition of macrophage tropism has been shown to result from internal changes to the FCoV S gene that develop spontaneously within an infected cat [41,42,43,44]. The exact nature of these mutations is still unknown. Previous investigations have suggested a correlation between amino acid substitutions M1058L and S1060A within the S protein and FIP [45,46]. Based on these results, a RT-qPCR that can distinguish FCoV with and without those mutations was developed in order to increase RT-qPCR specificity and subsequently, this S gene RT-qPCR was evaluated as a diagnostic test that is commercially available in different sample material from cats suspected of having FIP [47,48,49]. However, there is much controversy about the usefulness of the detection of S gene mutations as an addition to conventional 7b gene RT-qPCR [40,50,51,52]. S gene mutations could be detected in the CSF of cats with FIP previously [40,53], but the usefulness of CSF as sample material for S gene mutation analysis as a diagnostic tool has not been extensively studied.

Overall, appropriate diagnostic tests and material are lacking in cats suspected of having FIP without effusion, especially in those with neurological signs.

Therefore, it was the aim of this study to evaluate the usefulness of detecting FCoV RNA in CSF by RT-qPCR and additional S gene RT-qPCR as a diagnostic method in cats suspected of having FIP. Results of the two RT-qPCR should be compared to those of other diagnostic methods, such as cytology and immunocytochemistry (ICC).

## 2. Materials and Methods 

This prospective case-control study included 59 cats with clinical signs indicative of possible FIP. The study protocol was approved by the ethical committee of the Center for Clinical Veterinary Medicine of the LMU (reference number 52-27-07-2015). Of the 59 cats, nine cats had also been part of a previous study [32]. 

Cats were included based on the presence of one or more signs compatible with a clinical diagnosis of FIP, such as neurological signs, uveitis, effusion, fever (rectal temperature ≥40 °C), icterus or hyperbilirubinemia or hyperglobulinemia. In 30 cats, FIP was definitely confirmed. The cats had a mean age of 36 months (range 3–184 months). The age of two cats was unknown. Breeds included Domestic Shorthair (*n* = 25), Maine Coon (*n* = 2), Birman (*n* = 1), Chartreux (*n* = 1) and crossbred (*n* = 1). Sixteen of the cats were male (male intact *n* = 11, male neutered *n* = 5), 14 cats were female (female intact *n* = 8, female neutered *n* = 6). Diagnosis of FIP was established post mortem, either pathologically (macroscopically revealing effusions and/or yellow to white foci or nodules in different organs plus presence of typical histological lesions, including plasmacellular perivasculitis and/or accumulation of plasma cells accompanied by a fibrinous, necrotizing and pyogranulomatous inflammation; *n* = 18) or by histopathology and additional immunohistochemical (IHC) demonstration of FCoV antigen within macrophages in tissue lesions (*n* = 12). IHC of the brain was performed in 7/30 cats with FIP. Six of these cats with FIP had neurological signs and 24 cats suffered from non-neurologic FIP (Table 1). 

In the other 29 cats, FIP was considered an important differential based on the beforementioned signs, but a disease other than FIP, which explained the abnormalities, was definitely diagnosed. These 29 cats comprised the control group. The cats had a mean age of 111 months (range 3–210 months). The age of two cats was unknown. Breeds included Domestic Shorthair (*n* = 25), Persian (*n* = 2), Maine Coon (*n* = 1), and Somali (*n* = 1). Nineteen of the cats were male (male intact *n* =5, male neutered *n* = 14), 10 cats were female (female intact *n* = 2, female neutered *n* = 8). Eleven control cats had neurological signs (Table 2). Diagnosis of the respective disease was established either by histopathology after the cats died or were euthanized due to progression of their disease (*n* = 26), by cytology (when diagnosing neoplasia; *n* = 2) or by echocardiography (when diagnosing heart failure; *n* = 1). In 13/29 control cats, IHC for the presence of FCoV antigen in macrophages within affected organs was performed in addition and was negative. IHC of the brain was performed in 7/29 control cats.

CSF was obtained as surplus material in all of the cats (*n* = 59). Sampling was performed post mortem either by cerebellomedullary cisternal tapping or by direct transpallial aspiration of the lateral ventricles [32]. All CSF samples were stored at −80 °C until examination. The samples were analyzed within six months to four years.

RT-qPCR was performed in a commercial laboratory as described elsewhere [47]. Laboratory personnel was blinded with regard to the final diagnosis of the cats. After extraction of total nucleic acid, RT-qPCR was performed based on the FCoV 7b gene [54]. All 7b gene RT-qPCR-positive samples were examined by two additional RT-qPCR that targeted the M1058L and S1060A single nucleotide polymorphisms within the fusion peptide of the spike gene using highly specific fluorescent hydrolysis probes. Fluorescence intensity of a mutation probe exceeding twice that of the wildtype probe was considered positive for the presence of spike gene mutations. The outcome of the S gene RT-qPCR assay was defined based on the presence of mutated FCoV within a CSF sample. If mutated FCoV (corresponding to either amino acid substitution M1058L or S1060A) was detected in the sample, S gene RT-qPCR was considered positive. The presence of a mixed population of mutated and non-mutated FCoV within a sample was also considered positive. If no FCoV RNA was detected in a CSF sample, S gene RT-qPCR was considered negative. Likewise, S gene RT-qPCR was considered negative if only non-mutated FCoV was present within a CSF sample, if 7b gene RT-qPCR was positive but the virus load within a sample was too low (below 1.5 million viral RNA equivalents per ml of sample) to allow differentiation of mutated and non-mutated FCoV via S gene mutations, or if differentiation via S gene RT-qPCR failed despite a high viral load in the 7b gene RT-PCR. 

Additional diagnostic tests were cytological examination of the CSF and ICC to detect FCoV antigen within macrophages in seven cats with FIP and two control cats as described elsewhere [32]. Briefly, CSF was cytospun onto glass microscopy slides using a cytocentrifuge (Universal 16R, Hettich Zentrifugen, Tuttlingen, Germany) and stained with hematoxylin and eosin for cytologic examination using light microscopy. ICC was performed after cytocentrifugation using an avidin-biotin complex method (Vectastain, Vector Laboratories, Burlingame, CA, USA). A monoclonal anti-FCoV-antibody (clone FIPV3-70, LINARIS GmbH, Dossenheim, Germany) was used as primary antibody and diaminobenzidine-tetrahydrochloride as chromogen.

Sensitivity and specificity including 95% confidence intervals (95% CI) for the RT-qPCR and the detection of spike gene mutations were calculated. 

## 3. Results

FCoV RNA was detected by 7b gene RT-qPCR in the CSF from nine of the 30 cats with FIP: five cats with neurologic FIP (5/6, 83%) and four cats with non-neurologic FIP (4/24, 17%) (Table 1). S gene RT-qPCR identified mutated FCoV containing substitution M1058L in three of those nine cats: one cat with neurologic FIP and two cats non-neurologic FIP. In the remaining six cats, FCoV RNA was detected by 7b gene RT-qPCR but the virus load was too low to allow differentiation of mutated and non-mutated FCoV (Table 1 and Table 3). All 29 control cats had negative 7b gene RT-qPCR results (Table 2 and Table 3). 

Sensitivity and specificity including 95% CI of 7b gene RT-qPCR and the detection of spike gene mutations are shown in Table 4. Since FCoV RNA was not present in the CSF of any of the control cats, specificity of the S gene RT-qPCR could not be determined in this population of cats. When only cats with neurological signs were considered for analysis, sensitivity of 7b gene RT-qPCR increased markedly, whereas sensitivity of S gene RT-qPCR increased only mildly (Table 4).

ICC was performed in seven cats with FIP and paralleled the 7b gene RT-qPCR results in two cats, in which both assays were positive. S gene RT-qPCR demonstrated a low virus load in these two cats with positive 7b gene RT-qPCR and positive ICC. In one cat, 7b gene RT-qPCR was positive, but ICC was negative. S gene RT-qPCR showed a low virus load in this cat. In three cats, 7b gene RT-qPCR was negative, but ICC was positive, and CSF cytology was indicative of FIP (showing a pyogranulomatous inflammation) in two of these cats. IHC of the brain was available in 2/3 of the cats with discrepant RT-qPCR and ICC results and was negative in both cats. One cat had non-diagnostic ICC due to a lack of cellular material on the slide.

IHC of the brain was performed in 7/30 cats with FIP and paralleled the results of the 7b gene RT-qPCR in all but one cat, in which 7b gene RT-qPCR was negative, but IHC was positive. 

ICC was positive in one of the two control cats in which ICC of CSF was performed. CSF cytology was not strongly indicative of FIP in this cat, showing a lymphomonocytic inflammation, and IHC of brain tissue was negative.

## 4. Discussion

FIP has long been considered a uniformly fatal disease with a median survival time of only a few days [55]. In such a disease, specificity of a diagnostic test is more important than sensitivity, because it helps to prevent euthanasia secondary to misdiagnosis on the basis of false positive results. Very recently, novel antiviral drugs have demonstrated efficacy against experimentally induced and naturally occurring FIP, leading to recovery in a large number of affected cats and even those with neurologic FIP [23,24,25,26,27]. A highly promising approach involves GS-441524, a nucleoside analogue that leads to inhibition of viral RNA polymerase. In an initial field study, this drug was demonstrated to lead to clinical recovery and decrease in viral load in the majority of treated cats, but cats with neurological or ocular involvement were excluded [27]. More recently, however, a smaller case series could confirm efficacy even in cats with neurologic FIP [23]. Relapses were shown to occur in some cats in both studies. Nevertheless, since without these drugs, median survival time in cats with FIP was considered to be only about one week [56], such a prolongation of survival time is highly beneficial in cats with FIP and many cats even demonstrated sustained remission. These antiviral medications, however, are not yet commercially available for a broad range of cat owners and if they will become available, relatively high costs are likely. In any case, definitive ante mortem diagnosis using a test with high specificity is still important in cats suspected of having FIP in order to correctly define those cats that are appropriate patients for antiviral treatment and to avert financial threats from the owners. 

The results of the present study suggest a high specificity of 7b gene RT-qPCR in CSF of cats with neurologic FIP. Specificity of 7b gene RT-qPCR has previously been described as low for other sample material [40,47,52,57]. The absence of FCoV RNA in the CSF from cats of the control group could have several reasons. Cats might not have been infected with FCoV, as many control cats originated from single-cat households. In these, prevalence of FCoV infection is only 10–50%, in contrast to 80–100% in cats from catteries or other multi-cat households [58,59,60,61,62,63]. Another possible explanation for the lack of FCoV RNA in the CSF of a relevant number of control cats could be an intact BBB, which prevented viral leakage from the blood in the control cats. Nevertheless, eleven control cats had neurological signs and some of them even had inflammatory CNS disease, in which an impaired BBB can be expected.

Sensitivity of the 7b gene RT-qPCR in CSF was very low (30%) when all cats where analyzed, regardless of the presence of neurological signs and was even lower (17%) when only cats without neurological signs were considered. Post-mortem sampling and long storage time (even at −80 °C) causing viral or macrophage degradation is a possible reason for this low sensitivity. However, sensitivity was markedly higher (83%) in the group of cats with neurological signs. In fact, all but one cat with neurological signs were tested positive by 7b gene RT-qPCR. These results are comparable to previous case-control studies evaluating a conventional RT-PCR based on the FCoV 7b gene in CSF, which reported sensitivities of 21–42% that even increased to 31–86% when only cats with neurological signs were evaluated [16,39]. The higher sensitivity in cats with CNS involvement can be explained by a higher number of FCoV-infected macrophages present in the CSF. Still, it is interesting that FCoV RNA was present in the CSF also in a number of cats with non-neurologic FIP, and two cats had evidence of the presence of FCoV antigen detected by ICC despite the absence of neurological signs. This could result from a spillover of blood containing FCoV-laden macrophages across the BBB. This mechanism has been proposed for FCoV antibodies before [30]. Alternatively, some of the cats with FIP without neurological signs might have been in an early stage of developing CNS involvement. This was also discussed to be the reason for the presence of FCoV antigen in macrophages in the CSF from cats with non-neurologic FIP [32]. Since slight blood contamination was present in some of the samples, FCoV could have originated from blood in the cats without neurological signs. However, viral load is very low in blood [37] and thus, this possibility seems rather unlikely. 

When trying to detect mutations in the spike gene in those cats with positive 7b gene RT-qPCR, sensitivity decreased markedly (10% overall; 17% in cats with and 8% in cats without neurological signs). This could be expected by the general low virus load in blood and consequently in CSF samples, since the CSF is an extension of the blood compartment if the BBB is leaky. Additionally, storage of the samples, as mentioned before, might have influenced virus load. Therefore, some CSF samples with positive 7b gene RT-qPCR result but a low virus load had to be defined as negative for calculation of sensitivity of the S gene RT-qPCR, since the low virus load did not allow differentiation between mutated and non-mutated FCoV. This was the case in six CSF samples from cats with FIP. Similar findings have been made in studies using different other sample material, such as blood, effusion and tissue samples [40,52]. In these studies, sensitivity of a conventional RT-PCR decreased significantly when an additional step (such as sequencing or a second RT-PCR) was added after RT-PCR to differentiate between mutated and non-mutated FCoV, while specificity only moderately increased for tissue samples and even was unchanged for effusion or fluid samples (including effusion, CSF, and aqueous humor) [40,52]. Additionally, it is possible that low sensitivity of the S gene RT-qPCR resulted from mismatches between the used primers and the highly variable FCoV S gene. A recent study investigated the presence of spike gene mutations in FCoV from cats with and without FIP in Italy and suggested that sequencing of the regions of interest within the S gene might be more suitable for detection of S gene mutations than mutation analysis by using RT-qPCR with specific primers [46]. As a consequence of the low sensitivity of S gene RT-qPCR, it remains debatable whether the addition of S gene mutation analysis to RT-qPCR in CSF samples improves the ability to diagnose FIP and whether the S gene mutations examined are truly specific for FIP rather than being a marker of systemic spread of FCoV, as proposed previously [50]. Unfortunately, the results of the present study cannot clarify this any further, since no FCoV RNA was found in CSF of control cats. 

The S gene RT-qPCR used in this study has before been evaluated in different sample material, such as effusion, serum/plasma, aqueous humor or tissues [47,48,49,53]. Sensitivity was best in tissue (up to 71%) [48,53] and effusion (64–69%) [47,53], which is not surprising, given the fact that viral load in cats with FIP usually is high in these materials [37]. Nevertheless, FCoV RNA cannot always be detected in the effusion from cats with FIP. A recent study compared the detection of FCoV RNA by RT-qPCR and measurement of antibodies in effusion from cats with FIP and found negative results with both diagnostic tests, indicating that a combination of RT-qPCR and antibody measurement might be valuable to improve diagnostic sensitivity [64]. Sensitivity of the S gene RT-qPCR in aqueous humor was 13% and as such is comparable to the sensitivity in CSF in the present study [49]. It has been shown that the combination of different sample types can improve the ability to detect FCoV RNA by 7b and S gene RT-qPCR [53]. As a consequence, the overall diagnostic performance of the RT-qPCR assays might have been better if effusion, FNA samples of enlarged lymph nodes, spleen and liver and whole blood had been analyzed in addition to CSF.

Different diagnostic tests using the CSF were available and could be compared in some cats in this study. The combination of 7b and S gene RT-qPCR, ICC, CSF cytology and IHC of brain tissue was performed in six cats with FIP and two control cats. Discordant results were found in three cats with FIP, in which 7b gene RT-qPCR was negative, but ICC was positive. Interestingly, however, IHC of brain tissue was performed and was negative in two of these cats and none of the cats had neurological signs clinically. Thus, is could be assumed that ICC was false positive in these cats, maybe due to non-specific staining within the cytoplasm of macrophages. Nevertheless, upon regular cytology the CSF of both cats showed a pyogranulomatous inflammation, which was considered typical for FIP and therefore, it is also possible that these cats were on the verge of developing CNS involvement. One control cat with neurological signs had positive CSF ICC. 7b gene RT-qPCR was negative in this cat as in all other control cats. Most likely, this positive result can be explained by false positive ICC staining, possibly due to binding of the antibody to other cellular antigens than the FCoV nucleocapsid. Although it cannot ultimately be excluded that this control cat had FIP, CSF cytology and histopathology were not indicative of FIP and additionally, IHC of organ and brain tissue was negative. Thus, such scenario is highly unlikely.

This study had some limitations. The most important limitation, as mentioned before, is the fact that none of the control cats were positive for FCoV RNA in the CSF as determined by 7b gene RT-qPCR. As a result, it could not be evaluated whether S gene RT-qPCR was necessary or whether 7b gene RT-qPCR from CSF might be sufficient for the diagnosis of FIP in cats with or without CNS involvement. Second, only a limited number of cats with neurological signs were included. In a clinical setting, however, the CSF would most likely only be tapped if cats showed neurological signs. Therefore, sensitivity and specificity might be different in a group of cats that all have neurological signs and the results of the current study should be interpreted as preliminary results. However, since it could previously be demonstrated that FCoV antigen and RNA can be identified in the aqueous humor of a number of cats with FIP without ocular involvement [34,49], it was interesting to see whether the same was true for the CSF from cats with FIP without neurological signs. Thirdly, not all test (cytology and ICC of CSF and IHC of organ and brain tissue) were available for all cats, in part due to the fact that the amount of CSF that could be obtained from the cats was limited. This might raise the concern that misdiagnosis could have occurred in some cats. However, histopathology was consistent with the diagnosis and unambiguous in all cats with FIP and a disease other than FIP that explained the clinical signs was clearly diagnosed in all control cats. As a matter of fact, cats were only enrolled if they had clinical signs typical for FIP, which created a valuable study population that mimics the population of cats presented to veterinarians with a suspicion of FIP. Finally, in most cats of the study population, CSF was obtained post mortem and was stored for some time without preservatives, which might have influenced sensitivity of the RT-qPCR.

## 5. Conclusions

The results of the present study indicate that FCoV RNA can be detected by 7b gene RT-qPCR in cats with neurologic and non-neurologic FIP. Sensitivity of 7b gene RT-qPCR was high in cats with neurologic FIP, but low in cats without CNS involvement, indicating that further studies including cats with neurologic FIP should be conducted. However, when trying to detect spike gene mutations in RT-qPCR-positive cats, and only those with such mutations were considered positive, there was a decrease in sensitivity, but no increase in specificity (which was already 100%). This study therefore did not show a benefit of determining S gene mutations in addition to 7b gene RT-qPCR in the CSF.

## Figures and Tables

**Table 1:** Details of cats diagnosed with feline infectious peritonitis (FIP): clinical and/or laboratory signs upon presentation leading to inclusion, details of neurological signs, if present, final diagnosis and method of confirmation, results of the 7b and spike (S) gene realtime reverse transcriptase polymerase chain reactions (RT-qPCR), results of cerebrospinal fluid (CSF) cytology, immunocytochemistry (ICC), and immunohistochemistry (IHC) of affected organs and the brain, if available.

Cat	Clinical Presentation	Neurological Signs	Diagnosis	Method of Confirmation of Diagnosis	7b Gene RT-qPCR	S Gene RT-qPCR	CSF Cytology	ICC	Organ IHC	Brain IHC
1	Neurological signs	Ataxia, head tilt	FIP	IHC	Positive	Low ^1^	Pyogranu-lomatous	Negative	Negative	Positive
2	Neurological signs, fever, hyperglobulinemia	Head tilt	FIP	IHC	Positive	Low	Pyogranu-lomatous	Positive	Negative	Positive
3	Neurological signs, uveitis	Tetraparesis, nystagmus, anisocoria,	FIP	IHC	Positive	Low	Pyogranu-lomatous	Positive	Negative	Positive
4	Neurological signs, ascites	Ataxia, anisocoria	FIP	Histo- pathology	Positive	Low	n. a.	n. a.	n. a.	n. a.
5	Neurological signs, ascites, icterus, hyperbili- rubinemia	Seizures	FIP	Histo- pathology	Negative	n. a.	n. a.	n. a.	n. a.	n. a.
6	Neurological signs, fever, icterus	Ataxia	FIP	Histo- pathology	Positive	M1058L ^2^	n. a.	n. a.	n. a.	n. a.
7	Ascites, hyperbilirubinemia	No	FIP	IHC	Negative	n. a.	Pyogranu-lomatous	Positive	Positive	Negative
8	Hyperbilirubinemia, hyper- globulinemia	No	FIP	IHC	Negative	n. a.	Non- diagnostic	Non- diagnostic	Positive	Negative
9	Ascites	No	FIP	IHC	Negative	n. a.	Pyogranu-lomatous	Positive	Positive	Negative
10	Ascites, hyperbilirubinemia	No	FIP	IHC	Negative	n. a.	Few macrophages	Positive	Positive	n. a.
11	Ascites, icterus, hyperbilirubinemia, hyperglobulinemia	No	FIP	IHC	Positive	M1058L	n. a.	n. a.	Positive	n. a.
12	Ascites, pleural effusion, icterus, hyperbilirubinemia	No	FIP	Histo- pathology	Negative	n. a.	n. a.	n. a.	n. a.	n. a.
13	Ascites, fever, uveitis, hyperglobulinemia	No	FIP	Histo- pathology	Positive	Low	n. a.	n. a.	n. a.	n. a.
14	Ascites, icterus	No	FIP	Histo- pathology	Negative	n. a.	n. a.	n. a.	n. a.	n. a.
15	Ascites, hyperbilirubinemia	No	FIP	Histo- pathology	Negative	n. a.	n. a.	n. a.	n. a.	n. a.
16	Pleural effusion, fever, hyperglobulinemia	No	FIP	Histo- pathology	Negative	n. a.	n. a.	n. a.	n. a.	n. a.
17	Ascites, hyperbilirubinemia	No	FIP	Histo- pathology	Negative	n. a.	n. a.	n. a.	n. a.	n. a.
18	Pleural and pericardial effusion, hyperbilirubinemia	No	FIP	Histo- pathology	Negative	n. a.	n. a.	n. a.	n. a.	n. a.
19	Pleural effusion	No	FIP	Histo- pathology	Negative	n. a.	n. a.	n. a.	n. a.	n. a.
20	Ascites, fever	No	FIP	Histo- pathology	Negative	n. a.	n. a.	n. a.	n. a.	n. a.
21	Ascites, fever	No	FIP	Histo- pathology	Negative	n. a.	n. a.	n. a.	n. a.	n. a.
22	Ascites, icterus, hyperbilirubinemia, hyperglobulinemia	No	FIP	Histo- pathology	Negative	n. a.	n. a.	n. a.	n. a.	n. a.
23	Ascites, fever, icterus	No	FIP	Histo- pathology	Negative	n. a.	n. a.	n. a.	n. a.	n. a.
24	Ascites, icterus, hyperbilirubinemia	No	FIP	Histo- pathology	Negative	n. a.	n. a.	n. a.	n. a.	n. a.
25	Pleural effusion, fever, uveitis	No	FIP	Histo- pathology	Positive	Low	n. a.	n. a.	n. a.	n. a.
26	Fever, uveitis	No	FIP	Histo- pathology	Positive	M1058L	n. a.	n. a.	n. a.	n. a.
27	Ascites, icterus, hyperbilirubinemia, hyperglobulinemia	No	FIP	IHC	Negative	n. a.	n. a.	n. a.	Negative	Positive
28	Ascites, fever, icterus, hyperbilirubinemia	No	FIP	IHC	Negative	n. a.	n. a.	n. a.	Positive	n. a.
29	Ascites, fever, icterus, hyperbilirubinemia	No	FIP	IHC	Negative	n. a.	n. a.	n. a.	Positive	n. a.
30	Ascites, fever	No	FIP	IHC	Negative	n. a.	n. a.	n. a.	Positive	n. a.

^1^ Low = virus load too low to allow differentiation via S gene RT-qPCR. ^2^ M1058L = mutated FCoV with amino acid substitution M1058L or S1060A. n. a. = not available.

**Table 2:** Details of control cats: clinical and/or laboratory signs upon presentation leading to inclusion, details of neurological signs, if present, final diagnosis and method of confirmation, results of the 7b and spike (S) gene realtime reverse transcriptase polymerase chain reactions (RT-qPCR), results of cerebrospinal fluid (CSF) cytology, immunocytochemistry (ICC), and immunohistochemistry (IHC) of affected organs and the brain, if available.

Cat	Clinical Presentation	Neurological Signs	Diagnosis	Method of Confirmation of Diagnosis	7b Gene RT-qPCR	S Gene RT-qPCR	CSF Cytology	ICC	Organ IHC	Brain IHC
1	Neurological signs	Tetraplegia, head tremor	Globoid cell leuko- dystrophy	Histo- pathology	Negative	n. a.	Lymphomonocytic	Negative	Negative	Negative
2	Neurological signs	Seizures	Meningo- encephalitis	Histo- pathology	Negative	n. a.	Lymphomonocytic	Positive	Negative	Negative
3	Neurological signs	Ataxia, tail paralysis	Lymphoma, encephalomyelitis	Histo- pathology	Negative	n. a.	n. a.	n. a.	n. a.	n. a.
4	Neurological signs, icterus, hyper- bilirubinemia	Seizures	Necrotizing polio- Encephalo-pathy, hepatic lipidosis	Histo- pathology	Negative	n. a.	n. a.	n. a.	n. a.	n. a.
5	Neurological signs	Ataxia	Necrotizing leukomyelo-pathy, alimentary lymphoma	Histo- pathology	Negative	n. a.	n. a.	n. a.	Negative	Negative
6	Neurological signs	Seizures	Hippocampal sclerosis, renal lymphoma	Histo- pathology	Negative	n. a.	n. a.	n. a.	Negative	n. a.
7	Neurological signs	Seizures	Meningitis, cerebral edema, alimentary lymphoma	Histo- pathology	Negative	n. a.	n. a.	n. a.	Negative	n. a.
8	Neurological signs	Seizures	Meningo- encephalitis	Histo- pathology	Negative	n. a.	n. a.	n. a.	Negative	n. a.
9	Neurological signs, ascites, fever, hyper-globulinemia	Seizures	Multicentric lymphoma	Histo- pathology	Negative	n. a.	n. a.	n. a.	Negative	Negative
10	Neurological signs	Opistho-, pleurotho- tonus, seizures	Metastatic adenocarcinoma, ischemic encephalo- pathy	Histo- pathology	Negative	n. a.	n. a.	n. a.	n. a.	Negative
11	Neurological signs	Seizures	Limbic encephalitis	Histo- pathology	Negative	n. a.	n. a.	n. a.	n. a.	Negative
12	Pleural effusion	No	Sarcoma	Histo- pathology	Negative	n. a.	n. a.	n. a.	n. a.	n. a.
13	Ascites, pleural effusion	No	Renal lymphoma	Histo- pathology	Negative	n. a.	n. a.	n. a.	n. a.	n. a.
14	Hyper- bilirubinemia	No	Lymphoma	Histo- pathology	Negative	n. a.	n. a.	n. a.	n. a.	n. a.
15	Ascites, hyperbilirubinemia	No	Lymphoma	Histo- pathology	Negative	n. a.	n. a.	n. a.	n. a.	n. a.
16	Pleural effusion	No	Bronchial carcinoma	Histo- pathology	Negative	n. a.	n. a.	n. a.	n. a.	n. a.
17	Pleural effusion	No	Lymphoma	Cytology	Negative	n. a.	n. a.	n. a.	n. a.	n. a.
18	Pleural effusion	No	Pulmonary adeno- carcinoma	Histo- pathology	Negative	n. a.	n. a.	n. a.	n. a.	n. a.
19	Pleural effusion	No	Dilated cardio- myopathy, CHF	Echo- cardiography	Negative	n. a.	n. a.	n. a.	n. a.	n. a.
20	Ascites	No	Biliary adenocarcinoma	Histo- pathology	Negative	n. a.	n. a.	n. a.	n. a.	n. a.
21	Ascites	No	Lymphoma	Histo- pathology	Negative	n. a.	n. a.	n. a.	n. a.	n. a.
22	Icterus	No	Lymphoma	Histo- pathology	Negative	n. a.	n. a.	n. a.	n. a.	n. a.
23	Ascites	No	VSD, dextro- positioned aorta	Histo- pathology	Negative	n. a.	n. a.	n. a.	Negative	n. a.
24	Fever	No	CKD, pyelonephritis	Histo- pathology	Negative	n. a.	n. a.	n. a.	Negative	Negative
25	Pleural effusion	No	Hepatic carcinoma, pulmonary metastasis	Cytology	Negative	n. a.	n. a.	n. a.	n. a.	n. a.
26	Ascites, icterus	No	Biliary and pulmonary carcinoma	Histo- pathology	Negative	n. a.	n. a.	n. a.	Negative	n. a.
27	Ascites	No	Meso- thelioma, lymphoma	Histo- pathology	Negative	n. a.	n. a.	n. a.	Negative	n. a.
28	Ascites	No	End-stage CKD	Histo- pathology	Negative	n. a.	n. a.	n. a.	Negative	n. a.
29	Pleural effusion	No	Lymphoma	Histo- pathology	Negative	n. a.	n. a.	n. a.	Negative	n. a.

CHF = congestive heart failure; CKD = chronic kidney disease; n. a. = not available; VSD = ventricular septal defect.

**Table 3:** Summarized results of the spike (S) gene realtime reverse transcriptase polymerase chain reaction (RT-qPCR) in cats with feline infectious peritonitis (FIP) and control cats.

Group	Mutated FCoV (M1058L) ^1^	Mutated FCoV (S1060A) ^1^	Non-Mutated FCoV	Mixed FCoV ^2^	Low ^3^	High ^4^	Negative ^5^	Total
FIP	3	0	0	0	6	0	21	30
Controls	0	0	0	0	0	0	29	29

^1^ M1058L = mutated FCoV corresponding to amino acid substitution M1058L or S1060A; defined as positive in S gene RT-qPCR for statistical analysis. ^2^ Mixed FCoV = mixed population of mutated and non-mutated FCoV; defined as positive in S gene RT-qPCR for statistical analysis. ^3^ Low = positive 7b gene RT-PCR but virus load too low to allow differentiation; defined as negative in S gene RT-qPCR for statistical analysis. ^4^ High = positive 7b gene RT-PCR but differentiation not possible despite a high virus load; defined as negative in S gene RT-qPCR for statistical analysis. ^5^ Negative = negative 7b and S gene RT-PCR; defined as negative in S gene RT-qPCR for statistical analysis. FCoV = feline coronavirus.

**Table 4:** Sensitivity and specificity of the 7b and spike (S) gene realtime reverse transcriptase polymerase chain reactions (RT-qPCR). S gene RT-qPCR was considered positive if mutated feline coronavirus (FCoV) or a mixed population of mutated and non-mutated FCoV was present within a sample.

	All Cats (*n* = 59)		Cats with Neurological Signs (*n* = 17)		Cats without Neurological Signs (*n* = 42)	
	7b Gene RT-qPCR	S Gene RT-qPCR	7b Gene RT-qPCR	S Gene RT-qPCR	7b Gene RT-qPCR	S Gene RT-qPCR
Sensitivity (95% CI)	30.0% (16.5–48.0)	10.0% (2.7–26.4)	83.3% (41.8–98.9)	16.7% (1.1–58.2)	16.7% (6.1–36.5)	8.3% (1.2–27.0)
Specificity (95% CI)	100.0% (86.1–100.0)	n. d.	100.0% (70.0–100.0)	n. d.	100.0% (79.3–100.0)	n. d.

95% CI = 95% confidence interval; n. a. = not determined.

## Data Availability

Data is contained within the article.
